# Experimental realization of non-adiabatic universal quantum gates using geometric Landau-Zener-Stückelberg interferometry

**DOI:** 10.1038/srep19048

**Published:** 2016-01-07

**Authors:** Li Wang, Tao Tu, Bo Gong, Cheng Zhou, Guang-Can Guo

**Affiliations:** 1 Key Laboratory of Quantum Information, University of Science and Technology of China, Chinese Academy of Sciences, Hefei 230026, People’s Republic of China; 2 Department of Physics and Astronomy, University of California at Los Angeles, California 90095, USA

## Abstract

High fidelity universal gates for quantum bits form an essential ingredient of quantum information processing. In particular, geometric gates have attracted attention because they have a higher intrinsic resistance to certain errors. However, their realization remains a challenge because of the need for complicated quantum control on a multi-level structure as well as meeting the adiabatic condition within a short decoherence time. Here, we demonstrate non-adiabatic quantum operations for a two-level system by applying a well-controlled geometric Landau-Zener-Stückelberg interferometry. By characterizing the gate quality, we also investigate the operation in the presence of realistic dephasing. Furthermore, the result provides an essential model suitable for understanding an interplay of geometric phase and Landau-Zener-Stückelberg process which are well explored separately.

In quantum information science, a primary goal is to implement precise universal gates, because they provide the fundamental building blocks for constructing complex operations^1^. A universal set of quantum logic gates requires two types of non-commutable operations or arbitrary rotations around two axes on the Bloch sphere of a quantum bit (qubit). For fault-tolerant quantum computation, it is believed that an infidelity or error threshold ranging between 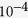 and 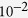 is required^2^,^3^,^4^,^5^; however, most experimental implementations thus far have fallen short of these thresholds^6^,^7^,^8^,^9^,^10^,^11^.

One promising approach towards this goal is to use quantum geometric phases which are acquired whenever a quantum system evolves cyclically along a path in the Hilbert space of quantum states^12^,^13^,^14^. In contrast to dynamical phases, geometric phases depend only on the geometry of the paths executed and are therefore resilient to certain types of errors^15^,^16^,^17^,^18^,^19^,^20^,^21^, which offers a reliable method to improve the fidelity of the gate operations. In the original proposal on geometric quantum computation^22^,^23^,^24^,^25^,^26^, if a system has multiple energy levels, a qubit is encoded in a doubly degenerate eigenspace. When the system evolves cyclically, it acquires not only a geometric phase factor but also undergoes a transition between the eigenstates in the degenerate subspace, which constitutes a set of universal unitary transformations for the qubit. In this technique, the system is typically changed adiabatically to guarantee the persistence of the degeneracy. However, the adiabatic condition makes any fast gate impossible, and consequently limits the application of the geometric gate operations in many quantum information tasks. A method for extending this process to non-adiabatic cases has been pursued^27^,^28^,^29^,^30^,^31^; however, the complex quantum control of the multi-level structure remains an experimental challenge.

In recent years, the Landau-Zener-Stückelberg (LZS) interference has become a powerful tool for studying two-level systems^11^,^32^,^33^,^34^,^35^. A new type of LZS interferometry has been proposed to have geometric fashion in a recent interesting letter^36^, and an initial experiment is reported^37^,^38^. Inspired by this original proposal^39^, here we joint the LZS interferometry and geometric quantum computation which are well explored separately in many quantum systems. We first outline the process in a general context^39^ and then present an experimental realization in a semiconductor quantum dot architecture. The results demonstrate how harnessing a well-controlled geometric LZS interferometry builds quantum gates for a two-level system, which combines the advantages of universality and speed. These types of geometric gates can thus be implemented conveniently in a wide variety of natural or artificial two-level systems.

Furthermore, in any realistic realization, a quantum system is influenced by its environment or control field leading to relaxation and dephasing during time evolution. The decoherence properties of geometric phase have been actively investigated in theory^15^,^40^,^41^,^42^,^43^,^44^,^45^,^46^,^47^. Therefore, there raises an interesting question about how an interplay of a geometric phase and a LZS dynamics manifests in the decoherence process. The present model can serve as a valuable tool to address this problem.

## Results

### Universal gates based on geometric phase for a two-level system

**Figure 1:**
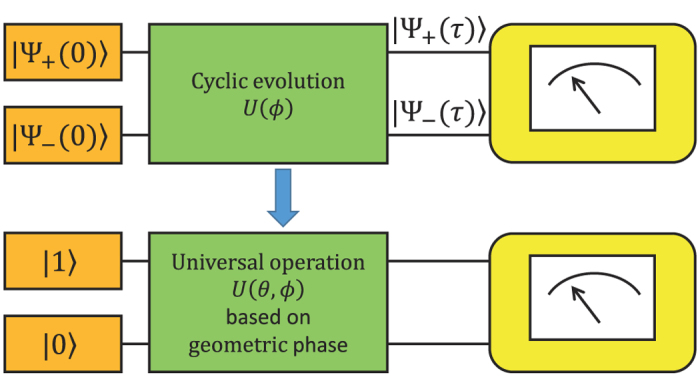
Quantum circuit for creation of cyclic evolution with a pair of orthogonal states 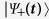 and 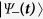, which is also equivalent to encoding the universal gate based on geometric phase in the computational basis states 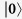 and 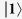

The principal idea is to generate a non-adiabatic, cyclic state evolution in a two-level system that results in a universal operation on the space spanned by the computational basis states, 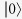 and 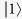 (Fig. 1)^39^. Let us write a state at 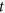 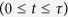 as 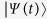. We consider a pair of orthogonal states 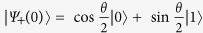 and 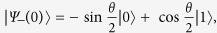 which act as auxiliary states and evolve cyclically after the gate operation. Here 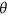 is the spherical coordinate of the state vector on the Bloch sphere. During the cyclic evolution, a phase 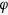 accumulates^13^, and the evolution operator can be expressed as follows: 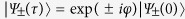 (see Supplementary Information for the definition of the phase factor during a cyclic evolution). Generally, this phase consists of both dynamical 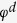 and geometric 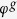 components. We note that in many schemes dynamical phase can also be acquired simultaneously in the cyclic evolution^15^. Specific operations or complex designs such as spin-echo technique can be used to remove the dynamical phase^15^,^39^, and the total phase reduces to pure geometric fashion. Here we focus on an interplay of dynamical phase and geometric phase.

For an arbitrary input state 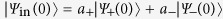, the final state is determined to be 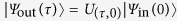, and the matrix representation of the final operator that acts on the basis states 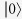 and 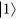 can be expressed as follows^39^:


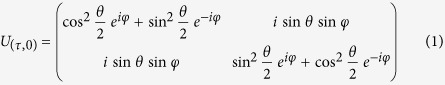


We can achieve a universal set of single qubit gates by selecting two non-commutable operations: 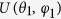 and 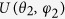.

### Electron qubit in a double quantum dot

**Figure 2:**
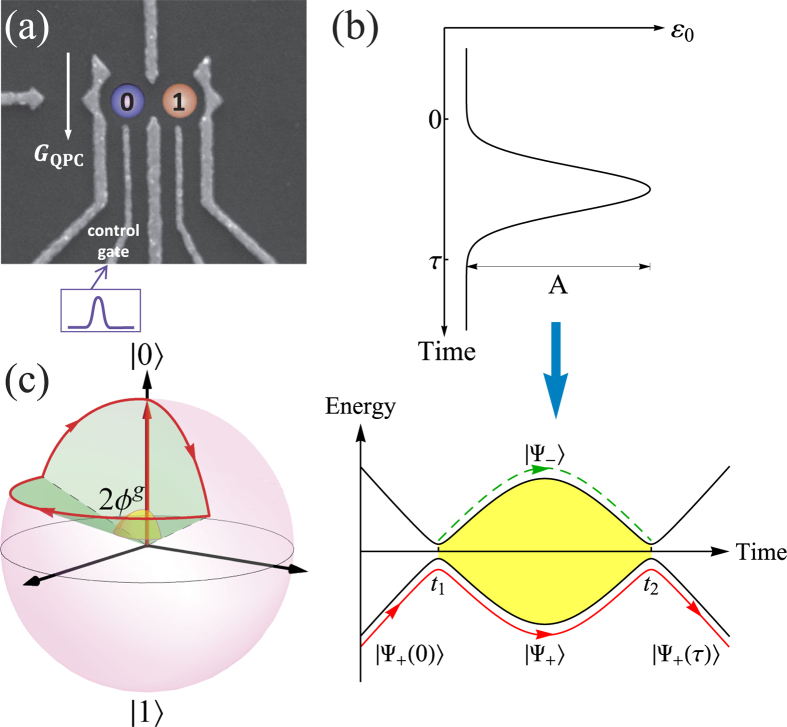
(**a**) Experimental setup for electron charge qubit in a double quantum dot. Scanning electron micrograph of the device that we used containing a double quantum dot and a nearby quantum point contact (QPC) sensor, with the locations of the states 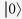 and 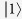 indicated by circles. The electric control pulse profile is sketched in the down part of the electrode. (**b**) Top: Schematic illustration of the control pulse form. The driving pulse 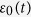 is implemented to vary the gate voltage of the dot and therefore the energy levels of the qubit. Down: The time evolution of the instantaneous energy levels. Driving the qubit from the initial state 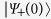 through the avoided crossing induces Landau-Zener transitions between the two paths. The paths recombine and interfere when the qubit is brought back through the avoided crossing. For destructive interference, the system returns back to the initial state, i.e., a cyclic evolution for the state 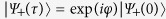. (**c**) Cyclic evolution represented on a Bloch sphere. The state space is the projected Hilbert space spanned by the basis states 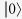 and 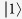. The state vector fulfils the destructive interference condition and evolves cyclically under two Landau-Zener processes. The geometric phase 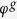 is determined by half of the solid angle swept by the evolution loop.

We describe an experiment conducted on an individual two-level system in a semiconductor electronic circuit (Fig. 2a, see Methods section for the details of devices and experimental techniques)^48^,^49^. An excess valence electron in the left and right quantum dots defines the charge occupation states 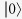 and 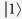. The charge qubit in a double quantum dot is typically regarded as a two-level system in the basis of 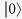 and 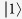. The Hamiltonian can be expressed as follows^48^,^50^,^51^,^52^:


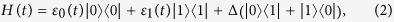


where 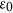 and 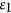 correspond to the discrete energy level in each dot; and Δ is the tunneling amplitude between the neighboring dots. In practice, the control pulse can be applied to vary the electrode voltages of the dots (for example, the left dot in our experiment), which leads to a change in the energy levels. The current approach is based on the cyclic evolution of the two orthogonal states 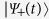 and 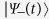, which are the instantaneous eigenstates of the system with energy eigenvalues 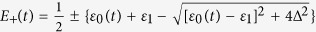 and 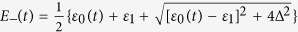, respectively. For our experiments, the anti-crossing gap 2Δ is fixed to 37.6 μeV and the reference level 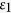 is set to zero. In this system, known as charge qubit, the two-level system is affected by its electromagnetic environment. We have experimentally determined an energy relaxation time of 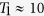 ns and a phase coherence time of 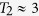 ns^48^.

### Realization of universal gates using geometric LZSM interferometry

Thus far, we have proposed a general method to achieve non-adiabatic universal quantum gates based on geometric phase accumulated by a two-level system. It is important to further consider implementing this technique with actual physical setups. This implementation can be achieved by driving the two-level system using a geometric LZS interferometry, with control parameters satisfying the condition of destructive interference.

The schematic diagram in Fig. 2b illustrates the energies of the instantaneous eigenstates of the qubit can be continuously tuned by the applied pulse 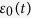. The envelopes 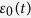 are Gaussian pulses with start point 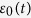, amplitude 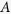 and total pulse length of 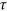. As indicated in Fig. 2b, the energy levels have a minimum distance of anti-crossing, and this minimum distance is realized at times 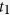 and 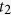. In the first stage, the control pulse takes the system from the initial state 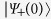 and passes through the anti-crossing point, at which a Landau-Zener transition occurs. The initial state is split into two occupied paths, one through 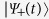 and the other through 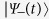, which is analogous to an optical beam splitter^33^,^35^. In the second stage, the control pulse takes the system back to the anti-crossing point, and the two paths can coherently interfere. Putting things together, the LZS interferometry can be treated as a successive unitary transformation between the initial state and the final state as follows^32^:


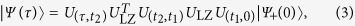


where 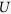 is the evolution matrix for each segment and 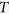 denotes a transposition of the matrix (the explicit expressions are provided in the Supplementary Information).

Typically, not every closed loop in the parameter space can result in a cyclic evolution in the Hilbert space of the LZS interferometry. The experiment is equivalent to an optical interferometer, where we have the interference of paths in phase space rather than in coordinate space. The interference phase^33^,^35^


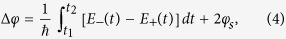


depends on the magnitude of the qubit energy detuning excursion for times 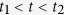 (shaded region in Fig. 2b), 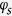 is the Stokes phase. The destructive interference corresponds to integer values of 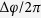
33,35. The destructive interference between the two transition paths, one through 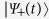 and the other through 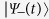 in the intermediate state, completely suppresses the probability to reach 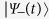 after the second crossing. Therefore the system undergoes an evolution of returning to the initial state, 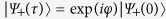, and this cyclic evolution loop corresponds to the operation 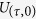 or 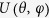 in the basis states 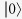 and 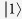.

**Figure 3:**
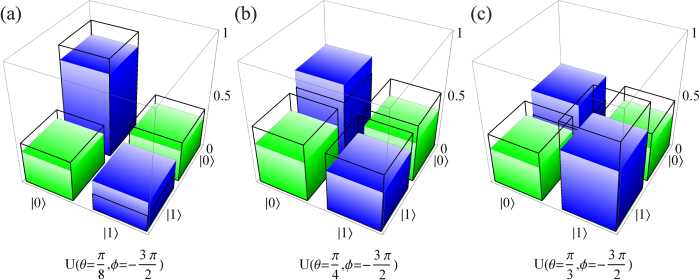
Characterization of the universal gates. Manhattan-style plots of the measured density matrices of the qubit states after the gate operations 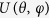: (**a**) 
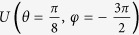
, (**b**) 
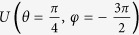
, (**c**) 
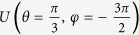
. The wire frames denote the theoretical values of ideal gates.

Different gates 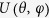 are achieved by adjusting the values of control parameters 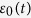, 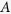 and 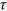 to satisfy the condition of destructive interference. The corresponding rotation angles of the gate operations are given by


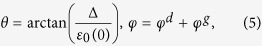


the dynamical phase 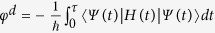; and an interpretation of the cyclic dynamics of the system is visualized in Fig. 2c, in which the value of the geometric phase 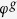 corresponds to half of the solid angle swept by the state vector on the Bloch sphere^13^,^39^,^53^ (see Supplementary Information for details on calculation of the dynamical and geometric phase). For examples, we evolve the pulse profiles 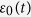 along three different loops, with the parameters 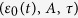 chosen respectively as (−45.2 μeV, 70.9 μeV, 257 ps), (−18.7 μeV, 27.2 μeV, 347 ps), and (−10.8 μeV, 15.4 μeV, 371 ps). The three gates results from these cyclic evolutions are denoted by 
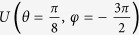
, 
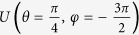
, and 
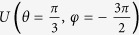
. The performance of the gates for three typical transformations is characterized by measured state tomography in Fig. 3.

### Dephasing during the geometric LZS process

**Figure 4:**
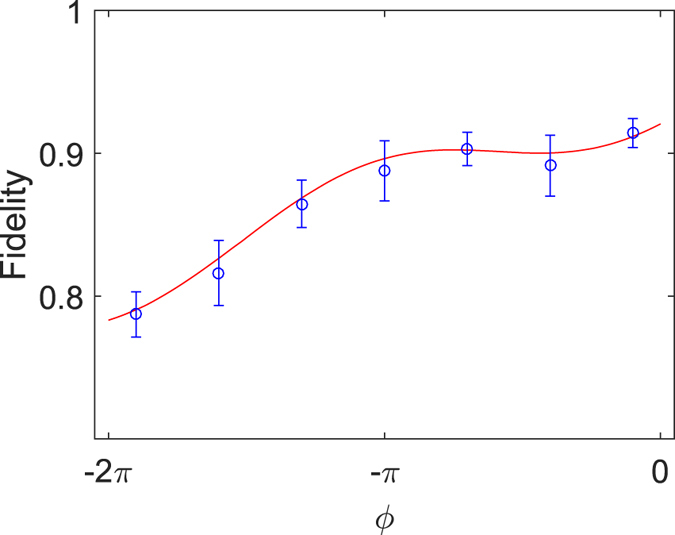
The fidelity *F* (blue circles) for the gates *U*(*θ, ϕ*), are provided as a function of the total phase *ϕ*, with *θ *=* *0. The fidelity is estimated from the state tomography of the final states. The red line corresponds to the theoretically calculated fidelity including the noise effect.

The experimentally obtained fidelity *F* of the gate process 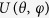 is provided in Fig. 4 as a function of total phase 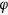, with 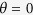. The fidelity is defined as the overlap between the physical and ideal density matrices after the gate operations (see Supplementary Information for theoretical calculations of the fidelity). One can also capture the essence of the observations through the intuitive picture presented in the following. Generally, fluctuations from control field cause errors in the acquired phase 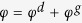 of the qubit. On one hand, if the fluctuations are sufficiently fast, the error in the geometric phase 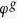 is minimum since the solid angle of the loop is preserved on average^15^,^18^,^20^,^42^,^44^,^53^. However, in our experiment, there is low-frequency fluctuations in the control field 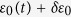 induced by charge noise coupling to the qubit^48^,^50^. Thus, the geometric phase 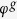 is sensitive to slow fluctuations, which cause the solid angle subtended by the path at the origin to change from one measurement to the next^18^,^44^,^53^. On the other hand, the dynamical phase 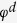 is dominated by Δ*ϕ* giving rise to the interference which is proportional to the duration of the accumulation process multiplied by the amplitude of the energy between two Landau-Zener tunneling points. The presence of the fluctuation field 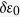 can change the position of the avoided level crossing and therefore cause fluctuations in 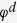
54. In the future, the influence of the geometric and LZS component on the quantum gates can be utilized and distinguished in a systematic way by using spin-echo technique to remove the dynamical phase^15^,^39^.

## Discussion

In summary, we realize a geometric LZS interferometry on a semiconducting quantum dot qubit using well-designed electric control pulse. We can achieve non-adiabatic gate operations that correspond to a representation of the complete SU(2) group, which is a central ingredient in geometric quantum computation. We can also investigate the fidelity of the resulting gate operations in the presence of realistic decoherence. Moreover, universal gates with inherent fault-tolerant geometric features demonstrated for semiconducting quantum devices can be implemented in general physical systems.

## Methods

The experiment was performed on a GaAs/AlGaAs heterostructure using a molecular-beam epitaxy, with a 95 nm deep two-dimensional electron gas (2DEG) with an electron density of 2.0 × 10^11^ cm^−2^ and a mobility of 6.0 × 10^4^ cm^−2^V^−1^s^−1^ at 4 K. The metallic (Ti-Au) surface gates were fabricated using electron-beam lithography. Figure 2a provides the scanning electron micrograph of the surface gates which shape the double quantum dot and a nearby quantum point contact (QPC) charge-sensing channel.

The device was cooled inside an Oxford Triton dilution refrigerator to a base temperature of 30 mK. To reduce charge noise, the sample was cooled while a positive voltage bias was applied to all the gates. The plunge gate was connected to the bias-tees, which enabled the application of DC as well as high frequency control voltage to this gate.

As a result of the capacitive coupling between the dot and the sensing quantum point contact (QPC), the record current through the QPC is increased or decreased when an electron moves from the left dot to the right or vice versa. Thus, the conductance change through the QPC with and without the manipulation pulse is used to determine the average charge occupation and converted to the reported probabilities^48^,^49^. Our experiments are performed for a number of different runs. Since they have shown identical results and physics, we present, for consistency, only the data collected from one run.

## Additional Information

**How to cite this article**: Wang, L. *et al.* Experimental realization of non-adiabatic universal quantum gates using geometric Landau-Zener-Stückelberg interferometry. *Sci. Rep.* **6**, 19048; doi: 10.1038/srep19048 (2016).

## Supplementary Material

Supplementary Information
