# Supplementary material for: Experimental realization of non-adiabatic universal quantum gates using geometric Landau-Zener-Stückelberg interferometry
Source: Sci Rep. 2016 Jan 7;6:19048. doi: 10.1038/srep19048 (PMC4703957; doi:10.1038/srep19048)
Supplement: Supplementary Information [file srep19048-s1.pdf]

Experimental realization of non-adiabatic universal quantum gates using geometric Landau-Zener-Stückelberg interferometry: Supplemental Information

Wang Li, Tu Tao, Gong Bo, Zhou Cheng, and Guo Guang-Can

I. CYCLIC EVOLUTION AND GEOMETRIC PHASE

In the method presented in this study, the quantum gates are implemented based on the geometric phase accumulated in the cyclic evolutions. Specifically, a normalized state $|\Psi(t)\rangle$ is cyclic in the interval $0 \leq t \leq \tau$ if and only if

$$|\Psi(\tau)\rangle = \exp(i\phi) |\Psi(0)\rangle , \tag{S1}$$

where ϕ is a real number [1–3]. In a cyclic evolution, there are dynamic and geometric contributions for a total phase of $\phi = \phi^d + \phi^g$. The non-adiabatic geometric phase ϕ^g can be associated with the evolution path in the project Hilbert space, which is known as the Aharonov-Anandan (AA) phase [1].

II. UNIVERSAL GATES BASED ON A PAIR OF ORTHOGONAL CYCLIC S-TATES

We focus on a two-level system (qubit) in the basis of $|0\rangle$ and $|1\rangle$. We consider a process in which a pair of orthogonal states $|\Psi_+(t)\rangle$ and $|\Psi_-(t)\rangle$ can evolve cyclically starting from $|\Psi_+(0)\rangle$ and $|\Psi_-(0)\rangle$. The two orthogonal states can be expressed as $|\Psi_+(0)\rangle = \cos\frac{\theta}{2}|0\rangle + \sin\frac{\theta}{2}|1\rangle$ and $|\Psi_-(0)\rangle = -\sin\frac{\theta}{2}|0\rangle + \cos\frac{\theta}{2}|1\rangle$. For a cyclic evolution, we can obtain the expression as follows:

$$|\Psi_\pm(\tau)\rangle = \exp(i\phi_\pm) |\Psi_\pm(0)\rangle ,$$

Furthermore, we can state the important relation of $\phi_+ = -\phi_-$ [3]. Thus, we can rewrite the cyclic evolution for $|\Psi_\pm(t)\rangle$ as follows:

$$|\Psi_\pm(\tau)\rangle = \exp(\pm i\phi) |\Psi_\pm(0)\rangle . \tag{S2}$$

Now, we can expand an arbitrary input state as $|\Psi_{in}(0)\rangle = a_+ |\Psi_+(0)\rangle + a_- |\Psi_-(0)\rangle$. A quantum logical gate is a unitary operator $U_{(\tau,0)}$ acting on the states of a qubit as $|\Psi_{out}(\tau)\rangle = U_{(\tau,0)} |\Psi_{in}(0)\rangle$, with $|\Psi_{out}(\tau)\rangle$ being the output state. In the computational basis of $|0\rangle$ and $|1\rangle$, we can express the operator as follows:

$$U_{(\tau,0)} = \begin{pmatrix} \cos^2\frac{\theta}{2}e^{i\phi} + \sin^2\frac{\theta}{2}e^{-i\phi} & i\sin\theta\sin\phi \\ i\sin\theta\sin\phi & \sin^2\frac{\theta}{2}e^{i\phi} + \cos^2\frac{\theta}{2}e^{-i\phi} \end{pmatrix} .$$

This is the single-qubit gate $U(\theta, \phi)$ in Eq. (1) of the main text. Generally, rotations about the arbitrary axes on the Bloch sphere corresponding to a representation of the complete

SU(2) group can be realized. To explicitly demonstrate the universal quantum gates, we realize three non-commuting operations or transformations in the main text.

III. STATE EVOLUTION OF A LANDAU-ZENER-STÜCKELBERG INTERFEROMETRY

A physical implementation of the general method is designed for a solid state two-level system. The Hamiltonian can be expressed as

$$H(t) = \varepsilon_0(t)\left|0\right\rangle\left\langle 0\right| + \varepsilon_1\left|1\right\rangle\left\langle 1\right| + \Delta(\left|0\right\rangle\left\langle 1\right| + \left|1\right\rangle\left\langle 0\right|), \tag{S3}$$

i.e., Eq. (2) of the main text. The current technique is based on the cyclic evolution of the two orthogonal states $|\Psi_+(t)\rangle$ and $|\Psi_-(t)\rangle$, which are the instantaneous eigenstates of the system with energy eigenvalues $E_+(t)$ and $E_-(t)$, respectively.

The manipulation pulse used to achieve the Landau-Zener-Stückelberg (LZS) interferometry for the qubit is illustrated in Figure 2b. In the adiabatic-impulse model [4], the system dynamics can be observed as a sequence of free evolutions and non-adiabatic transitions localized at the anti-crossing point. The calculation is more conveniently performed in the basis of $|0\rangle$ and $|1\rangle$.

The free evolution from time 0 to t_1 (or from t_2 to τ) can be described by a diagonal matrix with the form as follows:

$$U_{(t_1,0)} = \begin{pmatrix} \exp(-\frac{i}{\hbar}\int_0^{t_1} dt E_-(t)) & 0 \\ 0 & \exp(-\frac{i}{\hbar}\int_0^{t_1} dt E_+(t)) \end{pmatrix}.$$

Between t_1 and t_2 the propagator can be written as follows:

$$U_{(t_2,t_1)} = \begin{pmatrix} \exp(-\frac{i}{\hbar}\int_{t_1}^{t_2} dt E_+(t)) & 0 \\ 0 & \exp(-\frac{i}{\hbar}\int_{t_1}^{t_2} dt E_-(t)) \end{pmatrix}.$$

The Landau-Zener transition at the anti-crossing point can be described by a transfer matrix of the form as follows:

$$U_{LZ} = \begin{pmatrix} \sqrt{P} & -\sqrt{1-P}\exp(i\phi_s) \\ \sqrt{1-P}\exp(-i\phi_s) & \sqrt{P} \end{pmatrix},$$

where the Landau-Zener transition probability is given by $P = \exp(-2\pi\Delta^2/v\hbar)$; v is the sweep velocity of the driving pulse through the anti-crossing point; ϕ_s is the Stokes phase $\phi_s = \frac{\pi}{4} + \arg[\Gamma(1 - i\frac{\Delta^2}{v\hbar})] + \frac{\Delta^2}{v\hbar}(\ln\frac{\Delta^2}{v\hbar} - 1)$; and Γ is the Gamma function [4].

Hence, the LZS interferometry can be treated as a successive unitary transformation between the initial state and the final state as follows:

$$|\Psi(\tau)\rangle = U_{(\tau,t_2)}U_{LZ}^{T}U_{(t_2,t_1)}U_{LZ}U_{(t_1,0)}\,|\Psi(0)\rangle\,, \tag{S4}$$

i.e., Eq. (3) of the main text, where T denotes a transposition of the matrix.

Then, we can obtain the total transformation matrix as follows:

$$|\Psi(\tau)\rangle = \exp(-iS_+)\begin{pmatrix} K_{11} & K_{12} \\ K_{21} & K_{22} \end{pmatrix}|\Psi(0)\rangle\,, \tag{S5}$$

where the elements of the matrix can be calculated as follows:

$$\begin{aligned} S_+ &= \frac{\phi_+^2+\phi_-^2}{2}, S_- = \frac{\phi_-^2-\phi_+^2+2\phi_s}{2}, \\ K_{11} &= [P\exp(iS_-)+(1-P)\exp(-iS_-)]\exp(-i\phi_s-i2\phi_-^1), \\ K_{12} &= K_{21} = \sqrt{P(1-P)}[\exp(-iS_-)-\exp(iS_-)]\exp(-i\phi_-^1-i\phi_+^1), \\ K_{22} &= [P\exp(-iS_-)+(1-P)\exp(iS_-)]\exp(i\phi_s-i2\phi_+^1). \end{aligned}$$

Furthermore, the phase factors acquired during each stage of the process can be denoted as follows:

$$\phi_+^1 = \frac{1}{\hbar}\int_0^{t_1} dtE_+(t), \phi_-^1 = \frac{1}{\hbar}\int_0^{t_1} dtE_-(t), \phi_+^2 = \frac{1}{\hbar}\int_{t_1}^{t_2} dtE_+(t), \phi_-^2 = \frac{1}{\hbar}\int_{t_1}^{t_2} dtE_-(t). \tag{S6}$$

IV. DESTRUCTIVE INTERFERENCE AND CYCLIC EVOLUTION CONDITION

The key idea is to apply quantum gates or unitary transformations based on the cyclic evolutions, which is referred to the process where the state of the system returns to its original state after the gate operations. Typically, not every closed loop in the parameter space can result in a cyclic evolution in the Hilbert space.

In the following, we will illustrate that the LZS interferometry can support a cyclic evolution under the condition of destructive interference. In particular, we consider the initial state $|\Psi_+(0)\rangle = \begin{pmatrix} \cos\frac{\theta}{2} \\ \sin\frac{\theta}{2} \end{pmatrix} = \begin{pmatrix} 1 \\ 0 \end{pmatrix}$ where $\theta = 0$. Because the time evolution of the

state is explicitly provided in Eq. (S5), we find that the initial state can be transformed into the final state as follows:

$$|\Psi(\tau)\rangle = \exp(-iS_+) \begin{pmatrix} K_{11} \\ K_{21} \end{pmatrix}. \tag{S7}$$

The qubit state energy evolution between the recurrent Landau-Zener transitions provides a phase-space analog to the two arms and the beamsplitters of an optical Mach-Zehnder interferometer. $\Delta\phi$ is the phase difference between the two evolution paths [5, 6]: one through the state $|\Psi_+(t)\rangle$, and the other through the state $|\Psi_-(t)\rangle$,

$$\Delta\phi = 2S_- = \phi_-^2 - \phi_+^2 + 2\phi_s = \frac{1}{\hbar}\int_{t_1}^{t_2} dt[E_-(t) - E_+(t)] + 2\phi_s. \tag{S8}$$

Therefore the condition for the system returning to the initial state $|\Psi_+(0)\rangle$ after a period of evolution can be expressed as follows: $\Delta\phi = 2n\pi$, where n is a positive integer. This condition signifies that the two trajectories interfere destructively [5, 6] and enhance the probability of returning to $|\Psi_+(0)\rangle$ completely.

Consequently, the entire transformation operator reduces to a phase accumulation matrix, and Eq. (S7) can be rewritten as follows:

$$|\Psi(\tau)\rangle = \exp(i\phi)\,|\Psi_+(0)\rangle\,,$$

where $\phi = -(S_+ + \phi_s + 2\phi_-^1 + n\pi)$.

Based on Eq. (S6) and Eq. (S8), all the phase factors depend on the form of the control pulse $\varepsilon_0(t)$: initial position $\varepsilon_0(0)$, amplitude A and width τ of the control pulse. Thus, if a set of values of the control parameters are selected to satisfy Eq. (S8), the cyclic evolution condition is met in the LZS interferometry.

Next, if a geometric process is realized through the destructive interference in a LZS interferometry, one can use the transformation matrix $U_{(\tau,0)}$ or $U(\theta, \phi)$ as defined above to describe the operation results. There are three input control parameters: the initial position $\varepsilon_0(0)$, the amplitude A and the width τ of the driven pulse. There are two output result quantities: angles θ and ϕ which are functions of the above control parameters. One can link the input parameters and output quantities as equation (5) in the main text. Thus one can determine the input parameters for the fixed output quantities θ and ϕ to engineer the system evolution such as some desired gate operations. In general, it is a routine but lengthy job to exactly solve the equation (5) because of the nonlinearity and three numbers of input variables in the problem.

V. DYNAMICAL AND GEOMETRIC COMPONENTS IN THE TOTAL PHASE

The total phase ϕ acquired in the evolution contains both dynamic ϕ^d and geometric ϕ^g components. On one hand, the dynamical phase can be easily calculated as follows [1–3]:

$$\phi^d = -\frac{1}{\hbar}\int_0^\tau dt \left\langle \Psi(t)\right| H(t) \left|\Psi(t)\right\rangle . \tag{S9}$$

On the other hand, the geometric phase for a two-level system is most clearly visualized by a closed path in the projected Hilbert space [1–3, 7]. We define the polarization vector $\overrightarrow{n} = (\sin\Theta\cos\Phi, \sin\Theta\sin\Phi, \cos\Theta)$ on the Bloch sphere, which corresponds to the actual state evolution with a mapping relation $\overrightarrow{n}(t) = \left\langle\Psi(t)\right| \overrightarrow{\sigma} \left|\Psi(t)\right\rangle$. Thus, the geometric phase can be related to the solid angle subtended by the evolution curve on the sphere as follows [3]:

$$\phi^g = -\frac{1}{2}\int_C d\Phi(1-\cos\Theta), \tag{S10}$$

where C is a closed path on the Bloch sphere.

In particular, we consider the cyclic evolution from the initial state as $|\Psi_+(0)\rangle = |0\rangle$. Based on the successive unitary transformations in Eq. (S4), the cyclic evolution process can be described as follows. In the first stage from time 0 to t_1, the state vector $\overrightarrow{n}$ remains at the north pole. At a time t_1, the first Landau-Zener transition occurs, and the Bloch vector $\overrightarrow{n}$ moves along a meridian to the point $\overrightarrow{n}(t_1) = (\sin\Theta_1\cos\Phi_1, \sin\Theta_1\sin\Phi_1, \cos\Theta_1)$. In the second stage, $\overrightarrow{n}$ moves along the parallel to the point $\overrightarrow{n}(t_2) = (\sin\Theta_2\cos\Phi_2, \sin\Theta_2\sin\Phi_2, \cos\Theta_2)$, where $\Theta_2 = \Theta_1$. Then, at time t_2, the second Landau-Zener transition occurs, and the state vector returns to the north pole and remains there until τ. The path followed by $\overrightarrow{n}$ in the three-dimensional Bloch sphere is illustrated in Figure 2c of the main text.

When the state vector completes the closed circular path C, the geometric phase acquired by the state is $\phi^g = -\frac{\Theta_c}{2} = -(\phi_-^2 - \phi_+^2)(1-P)$, where Θ_c is the solid angle of the cone subtended by C at the origin.

In addition, it is interesting to study the effect of noise on the geometric phase and dynamical phase. On one hand, as illustrated in the Fig. S1 in the supplementary materials, the effect of fast noise on the geometric path can be averaged. However, slow noise can change the solid angle subtended by the path in the Hilbert space from one measurement to the next and cause error in the geometric phase. On the other hand, the noise will influence the

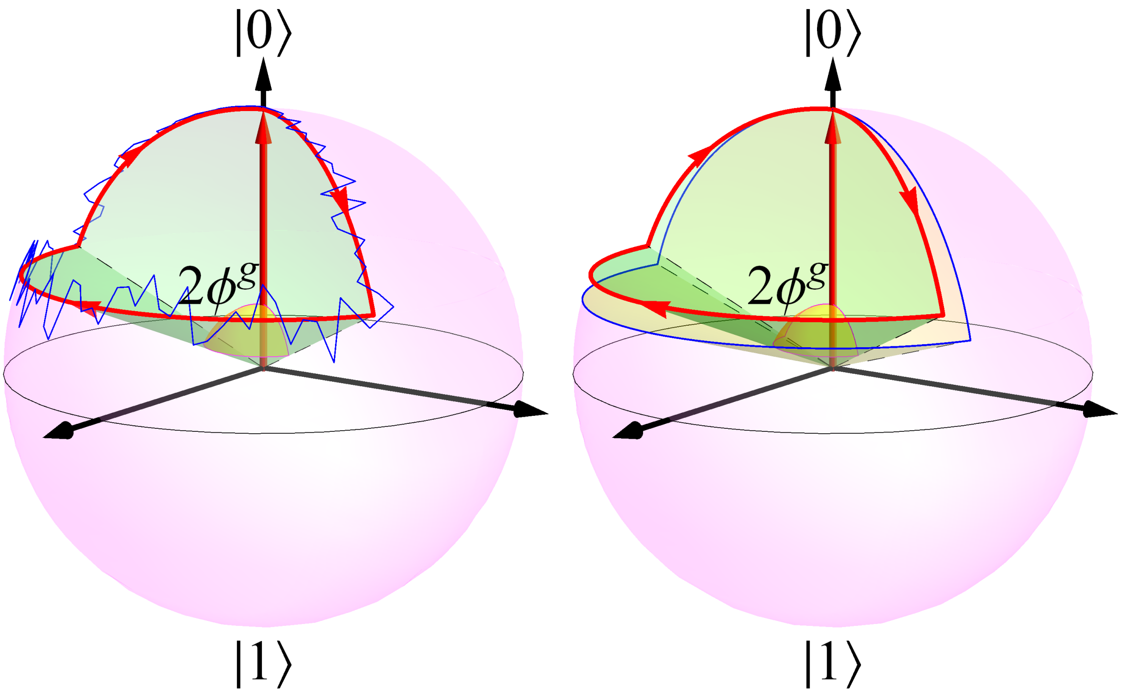

(a) (b)

FigS 1. Schematic figure of the noise effect on the geometric phase: (a) In the case of fast noise, the solid angle of the loop is preserved on average. (b) The slow noise change the solid angle enclosed by the path from one measurement to the next.

dynamical phase in a different way as discussed in the main text.

VI. TOMOGRAPHY AND FIDELITY OF THE GATE OPERATIONS

We fully characterize the gate operations using state tomography [8] which has been implemented in various systems: photons [9], superconducting qubits [10–12], respectively. In our experiment, a pulse repetition rate of 30 MHz was chosen to ensure that the qubit is relaxed to the initial state and to carry out a sufficient number of projective measurements (10^7 times) for an adequate signal-to-noise ratio. The ensemble averaging of these measurements, in terms of the average charge detector conductance, allows us to directly obtain the probability of the qubit states. These measurements allow for the evaluation of the qubit density matrix as shown in Figure 3 in the main text.

Furthermore, it is beneficial to quantify the performance of the operations and evaluate the impact of noise effects. Thus, we can estimate the fidelity of each gate operation. We can adopt a gate fidelity defined as follows [8, 13]:

$$F = \overline{\left|\langle\Psi(0)|\, U_P^{+} U_I \,|\Psi(0)\rangle\right|^2}.$$

where U_I is the ideal unitary transformation matrix of the gate operation; and U_P^+ is the Hermitian conjugate of the physical unitary transformation matrix. The definition can be naturally expressed as follows:

$$F = \mathrm{Tr}[\rho_P(\tau)\rho_I(\tau)]. \tag{S11}$$

where $\rho_P(\tau)$ and $\rho_I(\tau)$ are the physical and ideal density matrices after the gate operation, respectively; and Tr represents the trace.

VII. THEORETICAL MODELING AND SOLVING MASTER EQUATION

To construct a realistic model for our experiment, we find two main sources in the noisy environment [14]: one is electron qubit-phonon coupling and its contributions can be introduced by the dissipation rate parameters Γ_i (see below). The other is charge noise, well known to cause slow fluctuations of the electrical potential in semiconductor microstructures [15, 16]. Here we include the influence of the slow fluctuations by adding a noise term $\varepsilon_0(t) + \delta\varepsilon_0$ to the Hamiltonian H [17–20]. Usually it leads in ensemble average to an inhomogeneous, Gaussian broadening of width parameter σ_ε.

The time evolution of the qubit is given by solving the time-dependent master equations of the density matrix as follows:

$$\frac{d\rho}{dt} = -\frac{i}{\hbar}[\rho, H] + \frac{1}{2}\sum_{j=1}^{3}([L_j\rho, L_j^+] + [L_j, \rho L_j^+]), \tag{S12}$$

H is the Hamiltonian introduced in Eq. (S3), where the operators L_j are called Lindblad operators [8, 21, 22] and they describes the dissipative effect of the environment noise on the system in the Born-Markov approximation. For the case of a two-level system, the incoherent processes, include relaxation and pure decoherence processes:

$$L_1 = \sqrt{\Gamma_-}\sigma_-, L_2 = \sqrt{\Gamma_+}\sigma_+, L_3 = \sqrt{\Gamma_\varphi}\sigma_z. \tag{S13}$$

Here σ_- and σ_+ are spin ladder operators and σ_z is the Pauli z matrix. They correspond to relaxation from the excited state to the ground state with rate Γ_-, relaxation from the ground state to the excited state with rate Γ_+, and pure decoherence with rate Γ_φ.

In practice, we can simulate the evolution of the system by numerically solving Equation (S12), using the dissipation rate parameters for electron-phonon interaction and averaged over many realizations of the charge noise. Here we use the input parameters $\Gamma_{-} \approx 0.1$ GHz, $\Gamma_{\varphi} \approx 0.33$ GHz, and Γ_{-} has been set to zero, which are typical values consistent with the experimental measurement [18, 23]. Each run has a stochastic term $\delta\varepsilon_0$, which is sampled from a Gaussian distribution of the noise with width σ_{ε}. For low frequency charge noise, we use recent measurements of the standard deviation given as $\sigma_{\varepsilon} \approx 7.3$ μeV [18]. The results of calculated gate fidelity in the presence of realistic decoherece effect is provided in Figure 4 of the main text. We note that similar parameters and numerical analysis have been used in a variety of quantum dot settings [18–20].

---

[1] Aharonov, Y. & Anandan, J. Phys. Rev. Lett. **58**, 1593 (1987).

[2] Zhu, S. L. & Wang, Z. D. Phys. Rev. Lett. **89**, 097902 (2002).

[3] Zhu, S. L. & Wang, Z. D. Phys. Rev. A **67**, 022319 (2003).

[4] Shevchenko, S. N., Ashhab, S., & Nori, F. Phys. Rep. **492**, 1 (2010).

[5] Oliver, W. D. *et al.,* Science **310**, 1653 (2005).

[6] Petta, J. R., Lu, H., & Gossard, A. C. Science **327**, 669 (2010).

[7] Suter, D., Mueller, K. T., & Pines, A. Phys. Rev. Lett. **60**, 1218 (1988).

[8] Nielsen, M. A., & Chuang, I. L. *Quantum Computation and Quantum Information* (Cambridge Univ. Press, 2000).

[9] James, D. F. V., Kwiat, P. G., Munro, W. J., & White, A. G. Phys. Rev. A **64**, 052312 (2001).

[10] Liu, Y., Wei, L. F., & Nori, F. Europhys. Lett. **67**, 874 (2004).

[11] Steffen, M., *et al.* Science **313**, 1423 (2006).

[12] Rudner, M., *et al.* Phys. Rev. Lett. **101**, 190502 (2008).

[13] Zhu, S. L. & Zanardi. P. Phys. Rev. A **72**, 020301(R) (2005).

[14] Forster, F., *et al.* Phys. Rev. Lett. **112**, 116803 (2014).

[15] Petersson, K. D., Petta, J. R., Lu, H., & Gossard, A. C. Phys. Rev. Lett. **105**, 246804 (2010).

[16] Hayashi, T., Fujisawa, T., Cheong, H. D., Jeong, Y. H., & Hirayama, Y. Phys. Rev. Lett. **91**, 226804 (2003).

[17] Bylander, J., *et al.* Nat. Phys. **8**, 565 (2011).

[18] Dovzhenko, Y., *et al.* Phys. Rev. B **84**, 161302(R) (2011).

[19] Shi, Z., *et al.*, Phys. Rev. B **88**, 075416 (2013).

[20] Shi, Z., *et al.*, Nat. Commun. **5**, 3020 (2104).

[21] Lindblad, G. Commun. Math. Phys. **48**, 119 (1976).

[22] Breuer, H. P., & Petruccione, F. *Theory of Open Quantum Systems* (Oxford University Press, 2007).

[23] Cao, G., *et al.* Nat. Commun. **3**, 1401 (2013).
